# Visualization of chromosome condensation in plants with large chromosomes

**DOI:** 10.1186/s12870-017-1102-7

**Published:** 2017-09-12

**Authors:** Maria A. Kuznetsova, Inna A. Chaban, Eugene V. Sheval

**Affiliations:** 1 Belozersky Institute of Physico-Chemical Biology, Lomonosov Moscow State University, 119992 Moscow, Russia; 2 All-Russian Research Institute of Agricultural Biotechnology, Timiryazevskaja 42, 127550 Moscow, Russia; 3 LIA 1066 LFR2O French-Russian Joint Cancer Research Laboratory, 94805 Villejuif, France

**Keywords:** Mitosis, Chromosome, Condensation, Chromonema, Plants, Evolution

## Abstract

**Background:**

Most data concerning chromosome organization have been acquired from studies of a small number of model organisms, the majority of which are mammals. In plants with large genomes, the chromosomes are significantly larger than the animal chromosomes that have been studied to date, and it is possible that chromosome condensation in such plants was modified during evolution. Here, we analyzed chromosome condensation and decondensation processes in order to find structural mechanisms that allowed for an increase in chromosome size.

**Results:**

We found that anaphase and telophase chromosomes of plants with large chromosomes (average 2C DNA content exceeded 0.8 pg per chromosome) contained chromatin-free cavities in their axial regions in contrast to well-characterized animal chromosomes, which have high chromatin density in the axial regions. Similar to animal chromosomes, two intermediates of chromatin folding were visible inside condensing (during prophase) and decondensing (during telophase) chromosomes of *Nigella damascena*: approximately 150 nm chromonemata and approximately 300 nm fibers. The spatial folding of the latter fibers occurs in a fundamentally different way than in animal chromosomes, which leads to the formation of chromosomes with axial chromatin-free cavities.

**Conclusion:**

Different compaction topology, but not the number of compaction levels, allowed for the evolution of increased chromosome size in plants.

**Electronic supplementary material:**

The online version of this article (10.1186/s12870-017-1102-7) contains supplementary material, which is available to authorized users.

## Background

Most data about internal chromosome organization have been acquired from the studies of a small number of model organisms, the majority of which are mammals (human, mouse, Chinese hamster, etc.) that have relatively small genomes. The genome size in some plants and animals (e.g., in *Urodela*) is substantially larger than in mammals [1, 2]. The largest genome in plants that has been described is that of *Paris japonica* (1C = 152.23 pg) [3]. It is 50 times larger than that of a human monoploid genome (1C = 3.50 pg). As a general rule, the length of a haploid chromosome set is correlated with genome size [4]. Chromosome length is restricted by cell geometry [5], and therefore, an increase in chromosome width is necessary for an increase in chromosome size. The structural mechanisms that allow for an increase in chromosome width are poorly understood.

Inside chromatin, DNA is wrapped around nucleosomes, forming the ‘beads-on-a-string’ 10 nm chromatin fiber [6]. The interaction between the nucleosomes and linker histones leads to the formation of chromatin fibers with a diameter of 30 nm. Finch and Klug [7] demonstrated nucleosome fiber coiling in the presence of the H1 linker histone, which led to the chromatin organization that was referred to as the ‘solenoid’. Data in favor of a ‘zigzag’ nucleosome packaging were also presented [8, 9]. But recent data indicate that nucleosome mobility and the interaction of neighboring nucleosomes with each other leads to chromatin melting [10, 11], where 10 nm fibers are irregularly folded without the formation of a 30 nm chromatin fiber [10–15].

Analysis of chromosomes condensing during prophase and decondensing during telophase allowed for the description of additional fibrillar intermediates of chromatin folding: a 100–130 nm chromonema fiber and a 200–250 nm fiber [16]. Chromonemata were visualized in the partially decondensed chromosomes of prophase and telophase cells fixed in situ [17–22]. Recently, thick (approximately 70 nm) fibers, that seem to correspond to chromonemata, were visualized in *Drosophila* chromosomes by photoactivated localization microscopy (PALM) [23].

An approximately 200–250 nm folding subunit was also visualized within fully condensed animal metaphase chromosomes [24]. Analysis of chromosome condensation during the mitotic prophase showed that chromatids in early prophase have a diameter of 200–250 nm and that they were folded to form metaphase chromatids during late prophase [25]. Thus, in the case of animal chromosomes, it appears that the 200–250 nm fiber corresponds to the early prophase chromosome. The spatial topology of the 200–250 nm fiber is poorly investigated but some data indicate the absence of any regular, reproducible helical folding [26].

Chromosome condensation processes described for animal chromosomes are only one of the variants developed in the course of evolution [27, 28]. According to published data, mitotic chromosomes of some plants have specific morphological features that differentiate them from the intensively studied chromosomes of model animals and humans. In particular, the chromatin-free cavities in the axial region of the anaphase and telophase chromatids of some plants have been described using electron microscopy [17, 18, 21, 29, 30]. The cavities in the axial region have been seen in telophase chromatids of living cells of *Haemanthus katharinae* under phase contrast [31]. To our knowledge, these cavities have never been described in animal chromosomes. In contrast, after the in vitro [19, 32–34] or in vivo [35] decondensation of animal metaphase chromosomes, the densest chromatin is connected to the axial regions. It has been speculated that these cavities are the indication of either the existence of an additional level of chromatin condensation inside plant chromosomes or a different topology of chromatin fibers.

In this study, we analyzed chromosome condensation in plants with a large genome. We found that large chromosomes (average 2C DNA content exceeded 0.8 pg per a chromosome) in plants were organized differently than chromosomes in plants with small genomes. We visualized chromosome condensation intermediates in *Nigella damascena*, a plant with a large genome (2C = 21.10 pg), using the combination of chromosome segment labeling and electron microscopy morphometry. Our data indicate that specific condensation topology, but not number of condensation levels, allowed *N. damascena* to evolve into an organism with an increased chromosome size.

## Results

### Axial chromatin-free cavities appear in plants with average 2C DNA content exceeded 0.8 pg per a chromosome

**Fig. 1:**
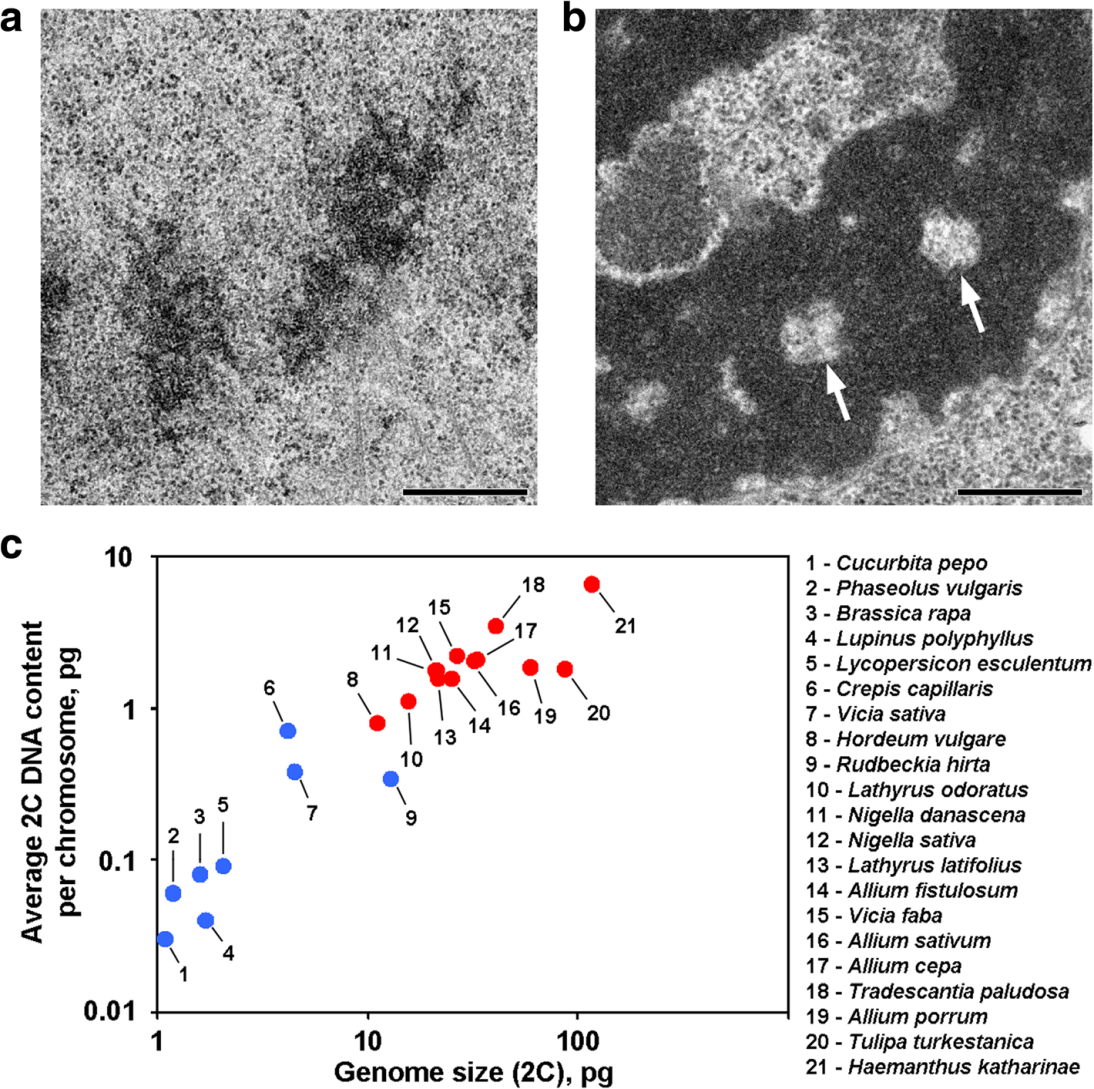
Two variants of chromosome organization in plants. **a** Telophase chromosomes of the common bean (*Phaseolus vulgaris*) as an example of chromosomes without axial chromatin-free cavities. **b** Telophase chromosomes of *N. damascena* with clearly visible axial chromatin-free cavities (arrows). **c** The presence of axial chromatin-free cavities depends on the genome and chromosome size. Blue dots represent plants in which chromosomes do not contain axial chromatin-free cavities; red dots represent plants in which chromosomes contain axial chromatin-free cavities. Scale bar: 0.5 μm

Anaphase and telophase chromatids of some plants contain chromatin-free cavities in the axial regions, which distinguish these plants from the majority of other plants and animals investigated to date (Fig. 1a, b). To our knowledge, reports describing such chromosome organization are restricted to plants with large genomes, and it can be assumed that such morphological organization is a specific feature for these plants. To ascertain this suggestion, we also analyzed anaphase and telophase chromatids from 12 species using electron microscopy (Table 1; Fig. 1a, b; Additional file 1: Figure S1). Figure 1c demonstrates that axial chromatin-free cavities were clearly seen only inside the chromosomes of plants with large genomes and with large chromosomes, but it seemed that average chromosome size correlated better with the presence or the absence of these cavities. Among plants with axial cavities, *Hordeum vulgare* had the smallest genome and the smallest average 2C DNA content per chromosome (0.79 pg). Thus, this chromosome organization is a hallmark of plant chromosomes whose 2C DNA content may be roughly estimated as exceeding 0.8 pg per chromosome.

**Table 1:** Chromatin-free cavities in anaphase/telophase chromatids of plants with different genome sizes

Plant	Genome size 2С, pg^a^	Number of chromosomes 2n^a^	Average chromosome size 2C/2n, pg	Axial chromatin-free cavity	Citation
*Cucurbita pepo* L.	1.10	40	0.03	–	Additional file 1: Figure S1a
*Phaseolus vulgaris* L.	1.20	22	0.06	–	Fig. 1a
*Brassica rapa* L.	1.60	20	0.08	–	Additional file 1: Figure S1b
*Lupinus polyphyllus* Lindl.	1.70	48	0.04	–	Additional file 1: Figure S1c
*Lycopersicon esculentum* Mill.	2.05	24	0.09	–	Additional file 1: Figure S1d
*Crepis capillaris* (L.) Wallr.	4.20	6	0.70	–	[18]
*Vicia sativa* L.	4.50	12	0.38	–	Additional file 1: Figure S1e
*Hordeum vulgare* L.	11.10	14	0.79	+	Additional file 1: Figure S1f
*Rudbeckia hirta* L.	13.00	38	0.34	–	Additional file 1: Figure S1 g
*Lathyrus odoratus* L.	15.55	14	1.11	+	Additional file 1: Figure S1 h
*Nigella damascena* L.	21.10	12	1.76	+	Fig. 1b
*Nigella sativa* L.	21.25	12	1.77	+	[18]
*Lathyrus latifolius* L.	21.75	14	1.55	+	Additional file 1: Figure S1i
*Allium fistulosum* L.	25.05	16	1.57	+	[18]
*Vicia faba* L.	26.65	12	2.22	+	[29, 30]
*Allium sativum* L.	32.45	16	2.03	+	[21]
*Allium cepa* L.	33.50	16	2.09	+	[21, 30]
*Tradescantia paludosa* E.S. Anderson & Woodson	41.25	12	3.44	+	[17]
*Allium porrum* L.	58.50	32	1.83	+	Additional file 1: Figure S1j
*Tulipa turkestanica* (Regel) Regel	87.00	48	1.80	+	[18]
*Haemanthus katharinae* Baker	117.70	18	6.5	+	[18, 31]

^a^Data from Plant DNA C-values Database [1]

### Chromosome condensation/decondensation can be visualized in semi-thin sections of *N. damascena* cells after 4′,6-diamidino-2-phenylindole (DAPI) staining

The most convenient object for the study of plant chromosomes, root apical meristem, does not allow for the acquisition of high-resolution images because of the out-of-focus fluorescence. Here, we used a method based on the application of semi-thin (200–250 nm) sections of roots embedded in LR White medium. The thickness of these sections was significantly lower than that of optical sections even if a confocal microscope was used. To find the morphological characteristics that allowed us to identify cells in different mitotic stages, we analyzed the morphology of cells stained with DAPI.

**Fig. 2:**
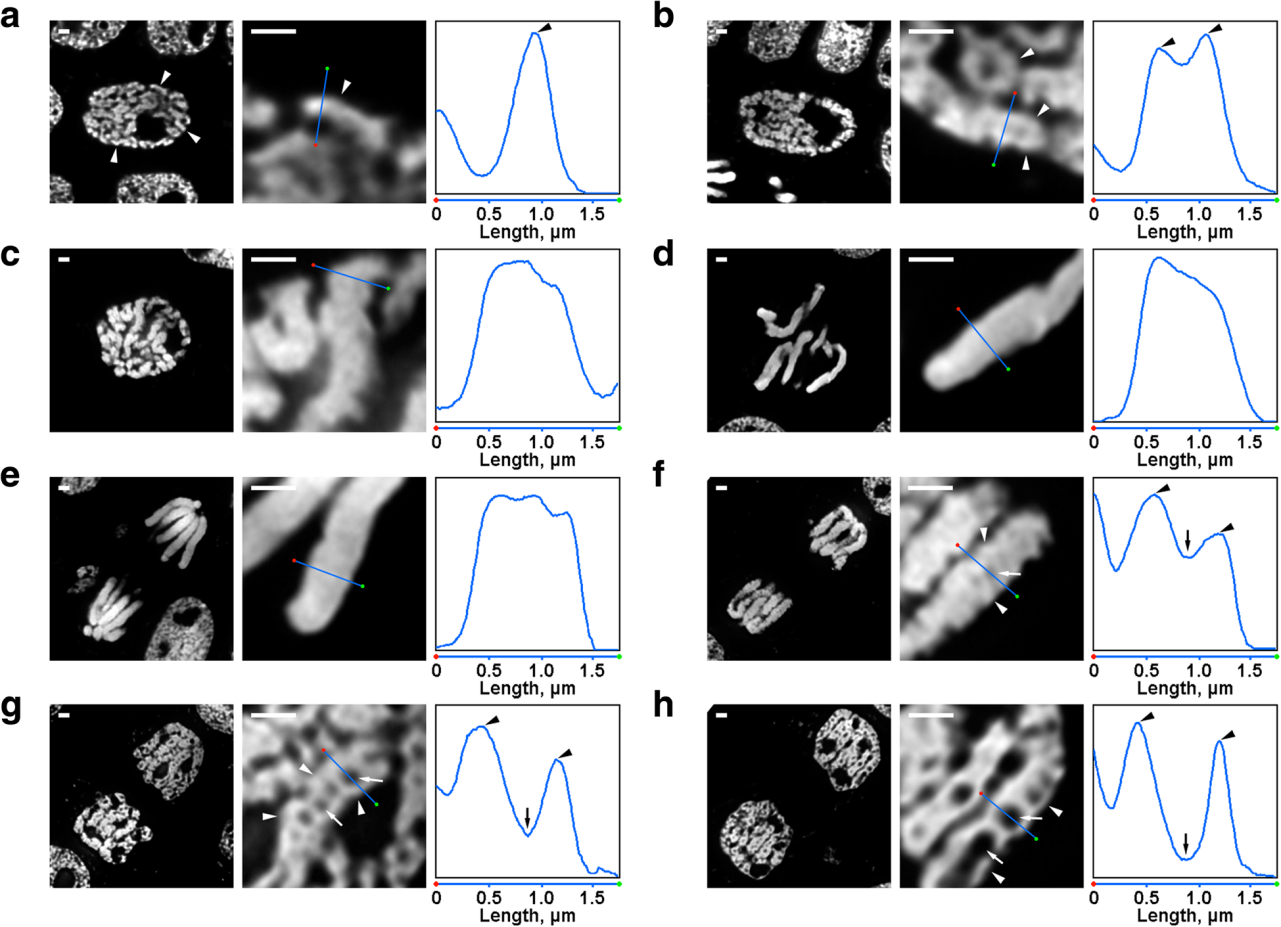
Morphology of the mitotic chromosomes of *N. damascena*. Left and central panels represent fluorescence microscopy images of DAPI stained semi-thin sections (general view and fragment); right panel represents a density plot through the line in the central panels. **a** Early prophase (chromosomes are indicated by arrowheads). **b** Middle prophase (fibers forming chromosomes, which seem to correspond to early prophase chromosomes, are indicated by arrowheads). **c** Late prophase. **d** Metaphase. **e** Anaphase. **f** Early telophase (axial chromatin-free cavities are indicated by arrows, fibers forming telophase chromosomes are indicated by arrowheads). **g** Late telophase. **h** G_1_-phase. Scale bars: 1 μm

There were several transformations of *N. damascena* chromosome organization that were easily visible even under the fluorescence microscope. Inside the early prophase nuclei, parts of thin chromosomes (approximately 0.6 μm in diameter) were visible (Fig. 2a). At middle prophase, thicker prophase chromosomes (approximately 1.2 μm) were formed, inside of which it was possible to distinguish the presence of thinner fibers, which probably corresponded to folded early prophase chromosomes (Fig. 2b). Late prophase (Fig. 2c), metaphase (Fig. 2d) and anaphase (Fig. 2e) chromosomes were densely condensed, and no internal organization was detected. During decondensation at telophase, the separation of chromatin fibers with a diameter of approximately 0.4 μm led to the appearance of chromatin-free cavities in the axial regions of chromatids (Fig. 2f), the size of which was gradually increased during telophase (Fig. 2g and h). The diameter of chromatin fibers in telophase chromosomes was comparable to the size of early prophase chromosomes. Thus, all mitotic stages of *N. damascena* were easily detected in semi-thin sections stained with DAPI.

### Chromosome condensation/decondensation during mitosis analyzed after 5-ethynyl-2′-deoxyuridine (EdU) incorporation

**Fig. 3:**
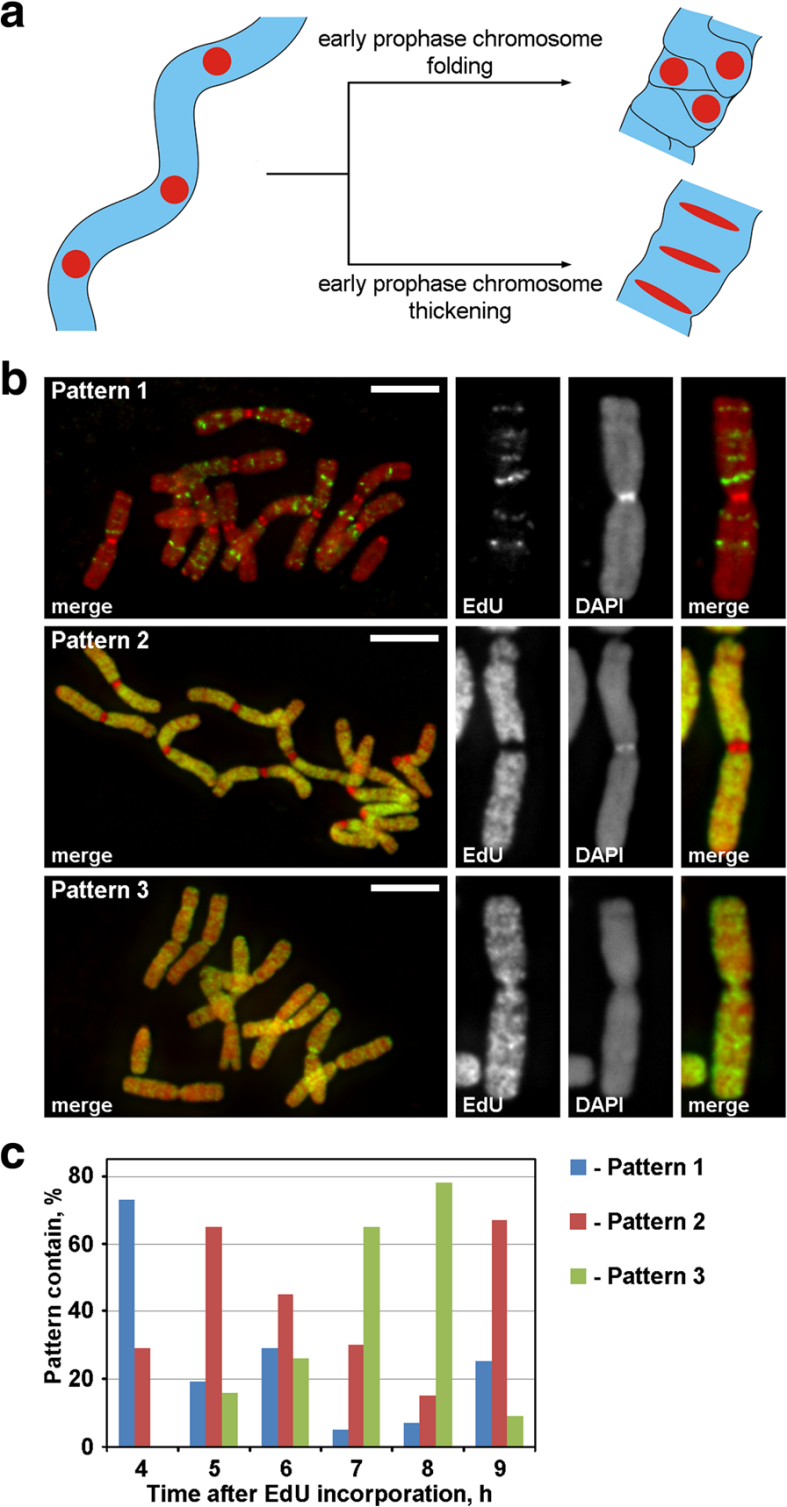
Chromosome labeling with EdU. **a** Labeled region localization and morphology revealed the principle of prophase chromosome condensation. Linearly arranged, labeled chromosome regions during the transition from early to late prophase would either lose the linearity arrangement (folding) or retain the linear arrangement (thickening). **b** Three patterns of EdU incorporation were detected in the chromosomes: labeling of discrete regions (pattern 1), labeling of chromosome arms but not centromeres (pattern 2) and labeling of both chromosome arms and centromeres (pattern 3). **c** Frequencies of different labeling patterns at different time points after EdU incorporation. Scale bars: 5 μm

During prophase, thin early prophase chromosomes were transformed into thick late prophase chromosomes. The thickening of prophase chromosomes may be a result of either early prophase chromosome folding or a result of its gradual thickening. To investigate these two possible mechanisms, it was necessary to mark the discrete chromosome foci that were linearly arranged inside the early prophase chromosomes and then analyze their spatial rearrangements during prophase condensation. As diagrammed in Fig. 3a, during the transition from early to late prophase, such foci would either lose the linear arrangement (folding) or would retain the linear arrangement and would stretch the foci into thin bands (thickening). To label the chromosome regions, we incorporated the synthetic nucleotide, EdU, which can be detected by click-chemistry, into chromatin during replication.

EdU was incorporated for 30 min into the roots of *N. damascena*, and after different chase periods (from 2 to 14 h), the chromosome spreads were made. Three patterns of chromosome labeling were detected: labeling of discrete regions (pattern 1), labeling of chromosome arms but not centromeres (pattern 2) and labeling of both chromosome arms and centromeres (pattern 3) (Fig. 3b). Pattern 1 was observed more often at 4 h after EdU incorporation, indicating that such labeling was typical for late S-phase (Fig. 3c; Additional file 2: Figure S2). In the case of pattern 1, the labeling of the homologous chromosomes was similar (Additional file 3: Figure S3), indicating a specific pattern of EdU incorporation. The chromosome arms were labeled during the rest of S-phase (patterns 2 and 3), but the centromeric regions were also labeled approximately at the boundary between early and late S-phase (pattern 3) (Fig. 3c).

**Fig. 4:**
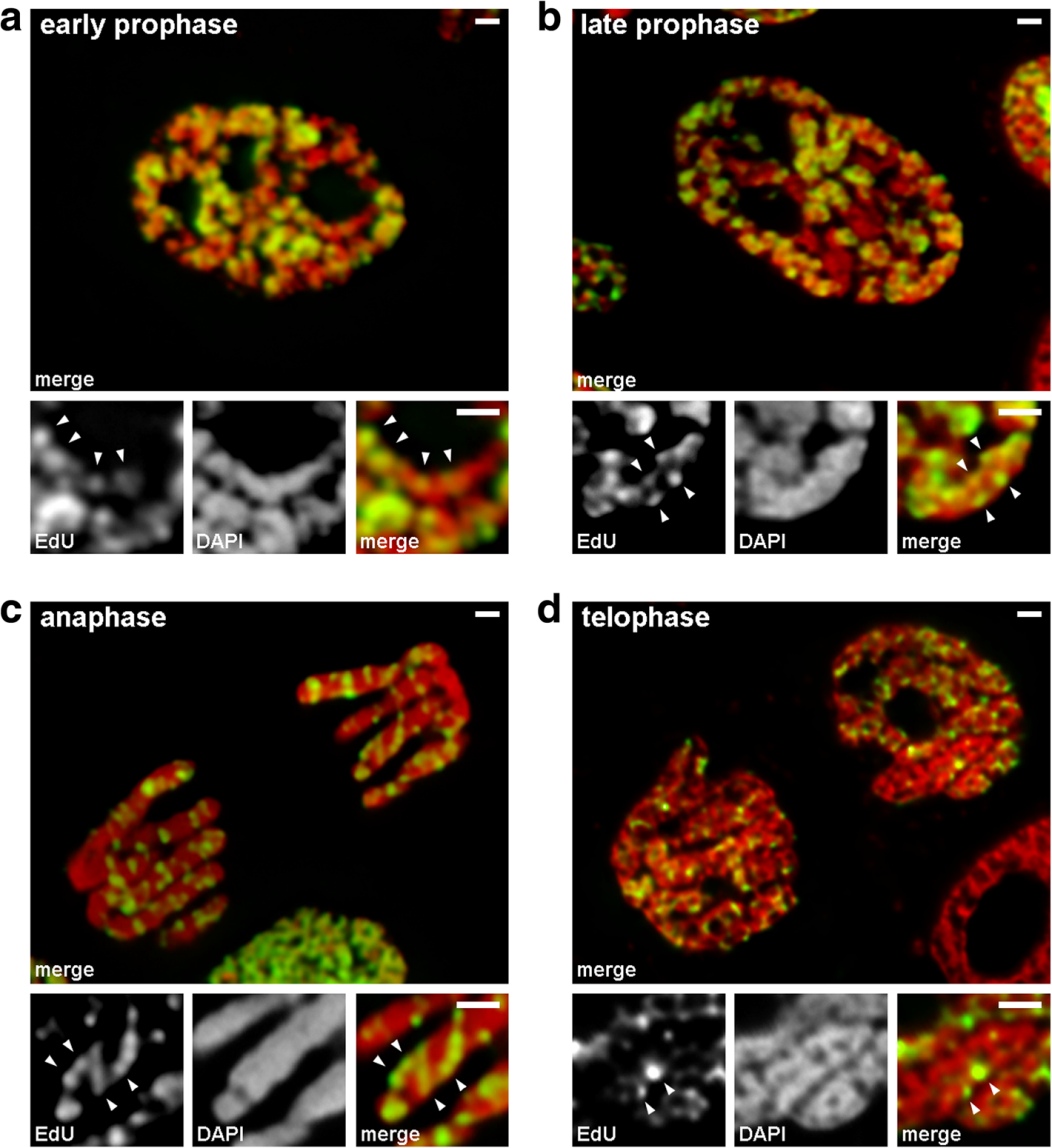
Chromosome condensation/decondensation during mitosis of *N. damascena* (mitotic cells whose chromosomes included EdU during late S-phase). **a** At early prophase, labeled regions were linearly distributed in thin chromosomes, spanning the chromosome width almost entirely. **b** At late prophase, labeled regions were scattered throughout the chromosome volume. **c** At anaphase, the labeling pattern was similar to that of late prophase chromosomes. **d** At late telophase, decondensation revealed thin fibers forming chromatids inside which labeled regions were distributed similarly to that inside early prophase chromosomes. Scale bars: 1 μm

We analyzed transitions from early to late prophase using chromosomes in which late-replicating chromatin was labeled (pattern 3). In early prophase chromosomes, the labeled regions were linearly distributed along thin chromosomes (Fig. 4a). In late prophase chromosomes, which were roughly twice as thick compared with early prophase ones, the labeled regions lost the linearity of distribution (Fig. 4b). This observation was not completely valid since prophase and metaphase chromosomes consist of two chromatids, which gradually segregate [36, 37], and this can lead to a loss in the linearity of the distribution of labeled regions. Therefore, we further analyzed anaphase chromatids (i.e., fully compacted chromosomes after chromatid segregation). In anaphase chromatids with a diameter approximately equal to the diameter of the late prophase chromosomes, the labeled chromatin regions were not linearly arranged but were distributed throughout the volume of the chromatids (Fig. 4c). At telophase, the chromatin-free cavities were clearly visible in the axial regions of the chromatids, allowing for detection of the chromatin fibers inside which the labeled regions were arranged linearly (Fig. 4d). Hence, during the transition from early to late prophase, the folding of early prophase chromosomes occurred. This result is in agreement with observations of DAPI-stained middle prophase chromosomes (Fig. 2b), inside which folded thin fibers were clearly visible.

### Chromosome condensation/decondensation during mitosis analyzed by electron microscopy

For the detection of the internal organization of chromatin fibers, the folding of which were described using light microscopy, we used electron microscopy. To detect and measure chromatin fibrillar substructures, we analyzed chromatin-free cavities, which separated chromatin fibers as we assumed (Additional file 4: Figure S4; Table 2).

**Table 2:** Chromosome and chromatin fiber characteristics in *N. damascena*

	Fiber diameter, nm (mean ± S.D.)			
Stage of mitosis	Interphase chromonema	Chromonema	‘300-nm fiber’	Chromosome/chromatid
Interphase	234 ± 49			
Preprophase		148 ± 30		
Early prophase		158 ± 46	527 ± 107^a^	
Late prophase			285 ± 102	809 ± 185
Metaphase				826 ± 120
Anaphase				895 ± 105
Early telophase		182 ± 47	422 ± 78	1038 ± 113
Late telophase	266 ± 54			979 ± 148
G_1_-phase	231 ± 55			

^a^At early prophase, ‘300 nm fiber’ corresponds to early prophase chromosome

**Fig. 5:**
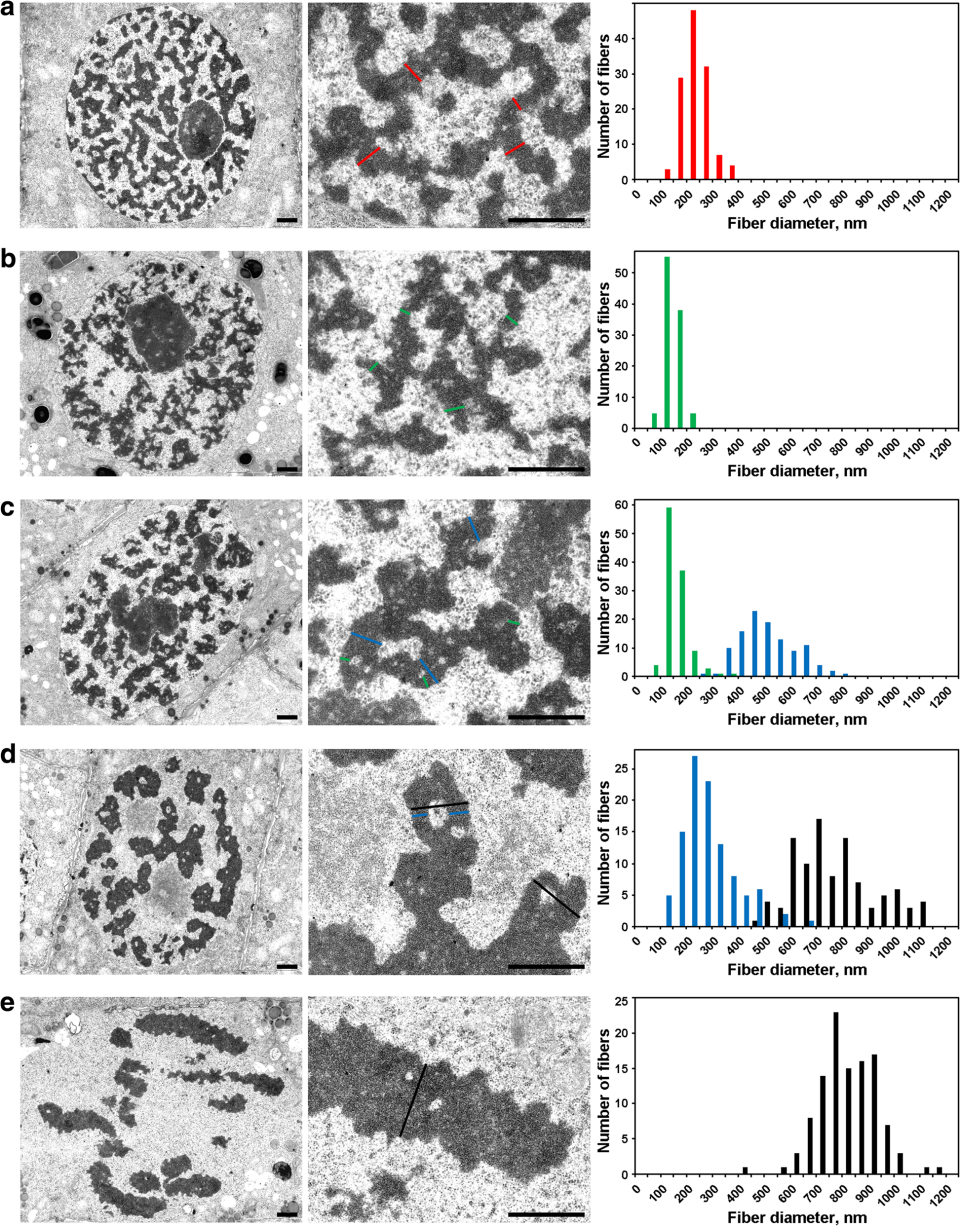
Electron microscopy morphometry of *N. damascena* chromosomes: chromosome condensation from interphase to metaphase. Left and central panels show ultrastructural organization (general view and fragment), right panels shows histograms depicting chromosome and chromatin fiber width distributions. **a** Interphase. **b** Preprophase. **c** Early prophase. **d** Late prophase. **e** Metaphase. The typical cross-sections of the chromosomes and/or chromatin fibrils are indicated with colored lines: red – interphase chromonemata (heterochromatin), green – chromonemata; blue – ‘300 nm fibers’; black – chromosomes. Scale bars: 1 μm

Interphase nuclei were filled with a meshwork of thick 234 ± 49 nm (mean ± S.D.) fibers (Fig. 5a), which may be referred to as ‘interphase chromonemata’, as discussed elsewhere [16]. Electron microscopy allowed for the detection of three stages of prophase chromosome condensation, the earliest of which was not possible to detect using light microscopy, and will be referred to here as ‘preprophase’. The second and the third stages corresponded to early and late prophase, as described above (Fig. 2a and c, respectively).

(1) At preprophase, the chromosomes were poorly separated from one another, and we could not accurately measure their diameter (Fig. 5b). Preprophase chromosomes were formed by fibers with a diameter of 148 ± 30 nm (Table 2), which most likely corresponded to the fibers with a diameter of 100–130 nm (chromonema), which are the main chromosome substructure of the prophase and telophase chromosomes in animal cells [18–20, 25, 38]. The data did not allow us to establish the identity of interphase and mitotic chromonemata, but previously reported data indicate that the interphase chromonemata are complexes formed by folded chromonemata [22].

(2) At early prophase, the separation of chromosomes occurred (Fig. 5c). The diameter of the chromosomes was 527 ± 107 nm, and these chromosomes were also formed by chromonemata with a diameter of 158 ± 46 nm.

(3) Finally, we analyzed late prophase cells, which contained thickened chromosomes with a diameter of 809 ± 185 nm (Fig. 5d). Some late prophase chromosomes contained cavities in the axial regions that allowed us to distinguish the fibers with a diameter of 285 ± 102 nm (‘300 nm fiber’). Analysis using the labeled chromosome segments demonstrated that early prophase chromosomes folded to form thicker late prophase chromosomes (Fig. 4). Therefore, it is possible to assume that the 285 ± 102 nm fibers and the 527 ± 107 nm early prophase chromosomes were the same fibers. The decreased diameter of the fibers may be associated with the chromatin compaction of early prophase chromosomes, which was also indicated by the disappearance of visible chromonemata.

**Fig. 6:**
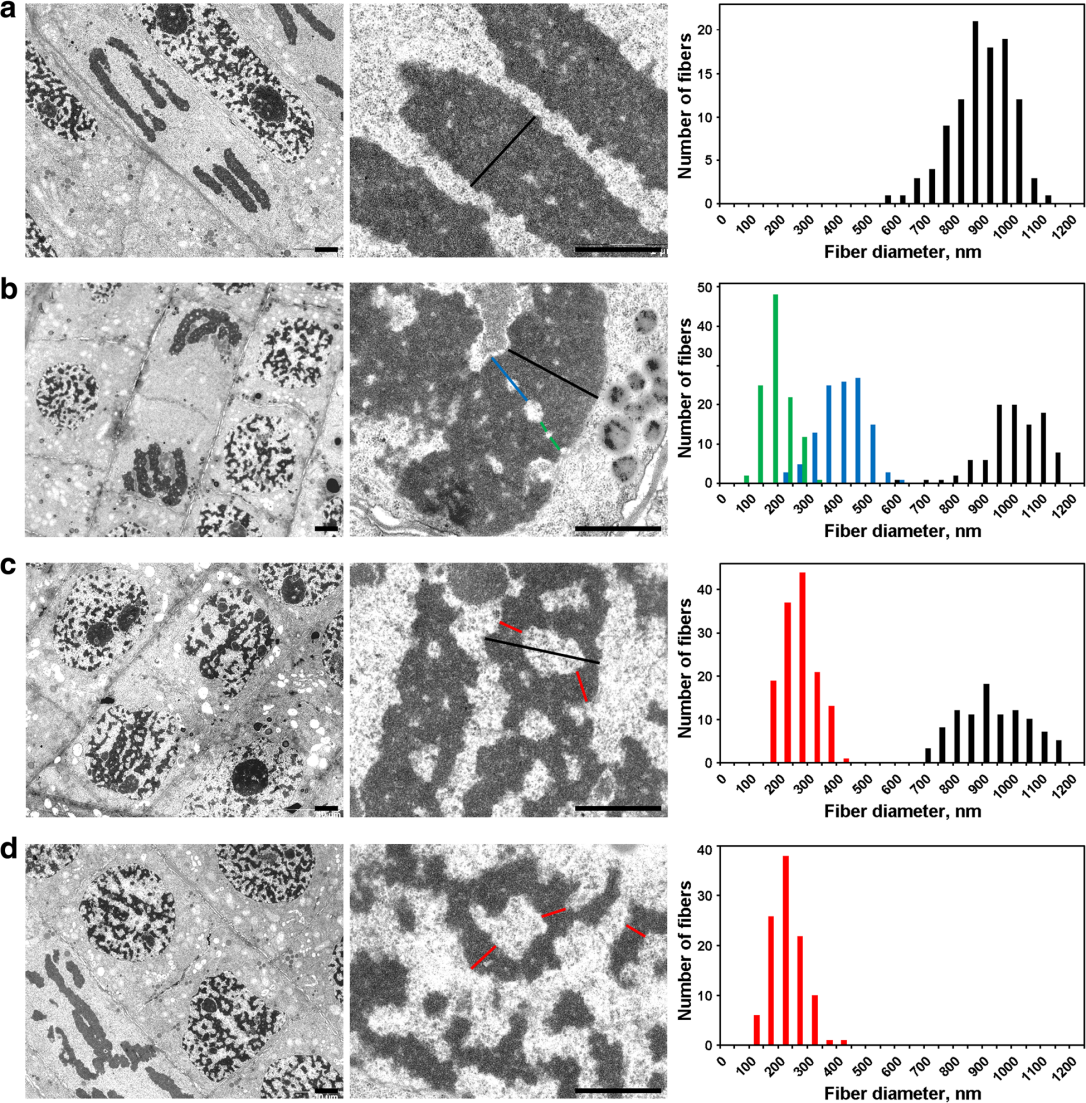
Electron microscopy morphometry of *N. damascena* chromosomes: chromosome decondensation from anaphase to G_1_-phase. Left and central panels show ultrastructural organization (general view and fragment), right panel shows histograms depicting chromatid and chromatin fiber width distributions. **a** Anaphase. **b** Early telophase. **c** Late telophase. **d** G_1_-phase. The typical cross-sections of the chromosomes and/or chromatin fibrils are indicated with colored lines: red – interphase chromonemata, green – chromonemata; blue – ‘300 nm fibers’; black – chromatids. Scale bars: 1 μm

Chromatin inside the metaphase chromosomes (Fig. 5e) and anaphase chromatids (Fig. 6a) was highly compacted, and, although rare chromatin-free cavities inside them were visible, an accurate distinction of any fibrillar substructures was impossible. In early telophase chromosomes, two types of cavities were clearly detected (Fig. 6b). Large cavities were located in the axial region of chromatids, allowing us to identify and measure fibers with a diameter of 422 ± 78 nm. In the central regions of these fibers, we detected small cavities that allowed for the identification of a second type of fiber with a diameter of 182 ± 47 nm. The former type of fibers might correspond to the ‘300 nm fibers’ inside late prophase chromosomes, the second type might correspond to the chromonemata.

At the late telophase (Fig. 6c) and G_1_-phase (Fig. 6d), the chromosomes were decondensed into chromosome domains in daughter nuclei where they cannot be easily observed. Nevertheless, it was possible to identify separated chromatid fragments. At this stage, the chromatids were formed by fibers with a diameter of approximately 250 nm, which roughly corresponds to the diameter of the interphase chromonemata. This observation in addition to the disappearance of chromonema fibers indicated that, at this stage of mitosis, chromatin fibers were refolded to form interphase complexes of condensed chromatin.

## Discussion

Chromosomes are too large for electron microscopy and too small for conventional light microscopy. Here, we used a combination of light and electron microscopies to study chromosomes in plants with large genomes. To improve the signal/noise ratio of light microscopy, we used a method of chromosome analysis on semi-thin (200–250 nm) sections of roots embedded in acrylic embedding medium. Similar approaches were developed previously [39, 40].

The anaphase and telophase chromatids of several plants have chromatin-free cavities in their axial regions [17, 18, 21, 29–31]; in contrast, animal chromosomes have an increased density of chromatin in the axial regions. Small cavities were described in plant metaphase chromosomes using scanning microscopy [41, 42]; the existence and size of these cavities depended on the preparation procedure [42]. Recently, it was demonstrated that a large portion of mitotic chromosomes is not composed of chromatin, and that periphery compartment comprises 30%–70% of the entire chromosome volume [43]. These estimations were done using cultured human cell, and one can assume that chromatin-free compartment in plant chromosomes may have not only peripheral localization.

Here, we used conventional aldehyde fixation, and small DNA-depleted cavities were also seen inside metaphase chromosomes. These cavities had a small size and irregular distribution, and therefore an accurate distinction of any fibrillar substructures inside metaphase chromosomes was impossible. The cavities inside prophase and telophase chromatids were larger and were easily detected using both light and electron microscopy. Of special importance, that cavities in the axial region have been seen in telophase chromatids of living cells of *Haemanthus katharinae* under phase contrast [31] indicating that these cavities were not an artifact of fixation.

We analyzed the published data and studied chromosomes of 12 plant species and found that such modified organization with axial cavities is specific to species with large chromosomes (the average 2C DNA content of 0.8 pg/chromosome is a threshold above which chromosomes change the chromosome condensation principle). It seems that this modified organization allowed plants to evolve the ability to increase chromosome width and, as a consequence, chromosome size. It should be stressed that such chromosome organization with axial chromatin-free cavities was detected in evolutionarily distant species, including both monocots and dicots, indicating that this organization might have evolved independently in different groups.

We separated two higher levels of chromatin condensation, chromonema and fibers that have a 285 ± 102 nm width at late prophase (i.e., in its maximally condensed state), and which can be referred to as a ‘300 nm fiber’. The latter fiber corresponded to chromosomes in early prophase. Apparently, these condensation levels correspond to the 100–130 nm fibers (chromonemata) and the 200–250 nm fibers described in animals.

Chromonema fibers were substantially wider in *N. damascena* chromosomes (approximately 150 nm) than those described in animal chromosomes [18–20, 25, 38], causing us to speculate that increased fiber width is one of the mechanisms that leads to chromosome enlargement. However, in *Allium cepa* prophase chromosomes, fibers with average diameters of 106 nm and 122 nm were detected [22], indicating that this may not be a universal characteristic of plant chromosomes.

The next level of fiber folding corresponded to early prophase chromosomes, similar to that described in mammal chromosomes [24, 25]. The diameter of these fibers was larger than that in mammal chromosomes, and the condensation from 527 ± 107 nm to 285 ± 102 nm was clearly visible during prophase. This condensation also led to the impossibility of detecting chromonemata inside condensed 300 nm fibers.

## Conclusion

**Fig. 7:**
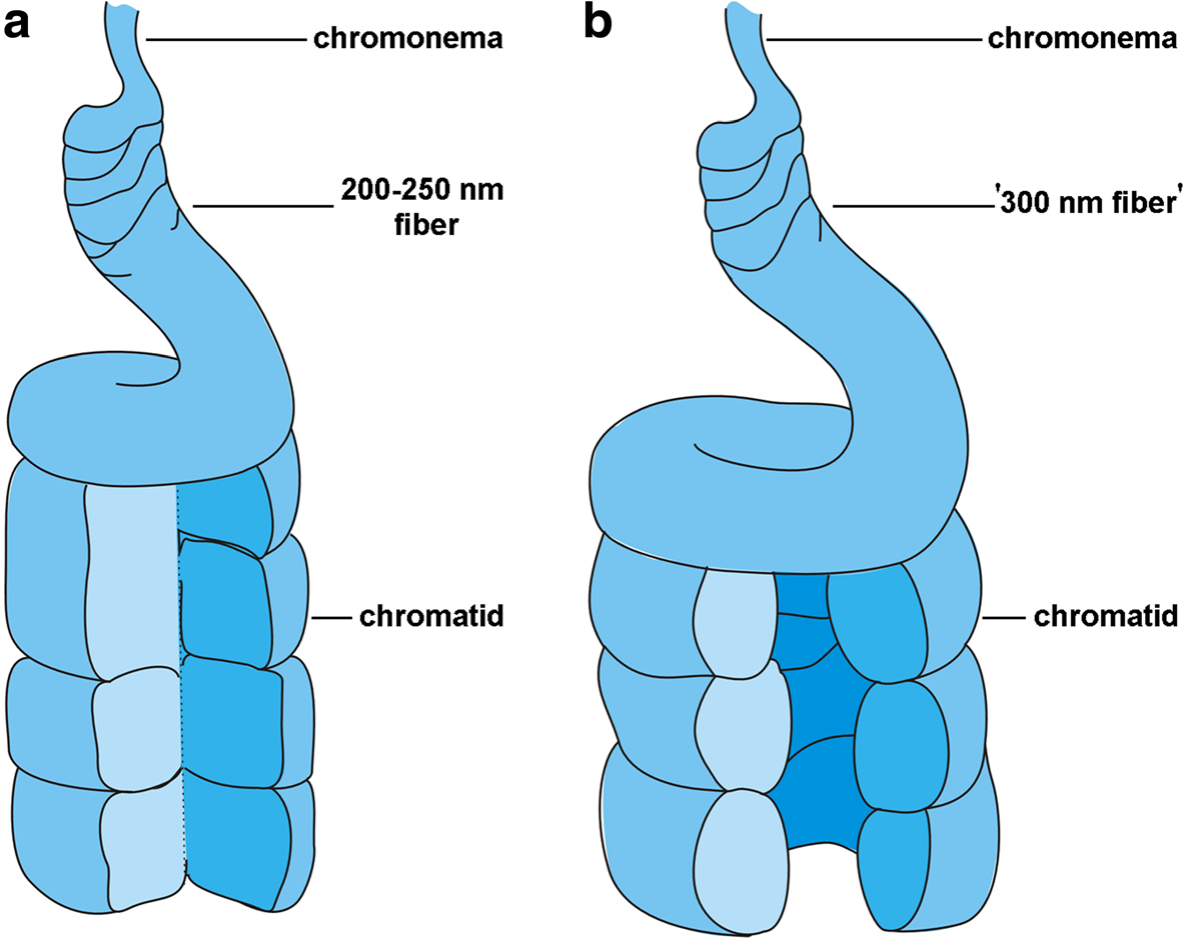
Models for chromosome condensation. **a** Chromatid organization in mammals. **b** Chromatid organization in plants with large chromosomes

Our results indicate that the folding topology of the ‘300 nm fiber’ in *N. damascena* was fundamentally different from that in mammals (Fig. 7). Although we cannot exactly define the topology of chromatin fibers in mitotic chromosomes, the peripheral location of ‘300 nm fibers’ was clearly seen in telophase chromosomes. Thus, increases in the size and diameter of *N. damascena* chromosomes are achieved preferentially by means of topology changes in the chromatin fibers rather than changes in the number of condensation levels.

## Methods

### Light microscopy

*N. damascena* seeds were purchased from Gavrish company (cat. no. 002364) (Russia). The seeds were grown in a Petri dish covered with dump filter paper at 25°С in the dark, and 10 mm long roots were used for the study. For chromatin labeling, the seeds were incubated in distilled water with 50 μM EdU (Invitrogen) for 30 min. For the chase experiments, seeds were labeled with EdU, were incubated in 200 μM thymidine (Sigma) for 30 min and then were grown in distilled water for 1.5–13.5 h (Additional file 2: Figure S2). Root tips that were 1.0 mm long were excised from roots and fixed in 2% paraformaldehyde in 0.5-fold PBS for 1.5 h. After fixation, the root tips were rinsed in PBS and embedded in LR White (Sigma, USA) according to the manufacturer’s protocol. Semi-thin sections were made using an Ultratome LKB-III (Sweden) and mounted on Formvar-coated cover glasses using a Perfect Loop (Ted Pella, Inc., USA). Slides were dried at 37°С for 2–3 h.

To obtain chromosome spreads, the EdU-labeled roots were fixed in a cold ethanol-acetic acid (3:1) solution. Root tips that were 1.0 mm long were cut off and rehydrated in solutions with decreasing concentrations of ethanol (96%, 70%, 50%, 30%, and 10%) for 5 min each. After washing in distilled water, the root tips were treated with an aliquot of enzyme solution composed of 0.10% pectolase, 0.15% cellulose and 0.10% cytohelicase (Sigma) in 10 mM citrate buffer and 0.1 mM EDTA for 65 min at 37°С. The root tips were squashed under a cover slip in a drop of 45% acetic acid; after freezing in liquid nitrogen, the cover slip was removed by razor.

EdU was detected using a Click-iT EdU Alexa 555 Imaging Kit (Invitrogen). DNA was stained with DAPI and mounted in Mowiol (Calbiochem) with the anti-bleaching agent, DABCO (Sigma). Image stacks were acquired using an Axiovert 200 M microscope equipped with a Plan-Apochromat 100/1.4 objective (Carl Zeiss) and a cooled CCD camera, ORCAII-ERG2 (Hamamatsu). Deconvolution was done according to the constrained iterative algorithm in the AxioVision 3.1 software (Carl Zeiss).

### Electron microscopy

Root tips were fixed in 4% glutaraldehyde in 0.1 M Sörensen’s phosphate buffer, post-fixed in 1% OsO_4_, dehydrated in ethanol and propylene oxide and embedded in Epon 812 (Fluka). The ultra-thin sections were stained with uranyl acetate and lead citrate and examined using a JEM-1400 electron microscope (Jeol). For morphometry and image analysis, ImageJ2 software was used (9–18 cells for each mitotic stage were analyzed). For the final presentation, the brightness and contrast of the images were corrected using Photoshop software (Adobe).

## Additional files

Additional file 1: Figure S1. Chromosome organization in plants with different genome sizes. a *Cucurbita pepo*; b *Brassica rapa*; c *Lupinus polyphyllus*; d *Lycopersicon esculentum*; e *Vicia sativa*; f *Hordeum vulgare*; g *Rudbeckia hirta*; h *Lathyrus odoratus*, i *Lathyrus latifolius*; j *Allium porrum*. Axial chromatin-free cavities are indicated by arrows. For each plant species, several anaphase and telophase cells were analyzed, and the image of the most representative cell is shown. Scale bars: 0.5 μm. (PDF 3315 kb)

Additional file 2: Figure S2. Asynchronously growing cells of *N. damascena* roots were pulse labeled with EdU for 30 min, incubated in 200 μM thymidine for 30 min, and then the incubation was continued in the distilled water (i.e., in the absence of EdU). The brief pulse labeled all S-phase cells, and the initial appearance of EdU-labeled mitotic figures thus denoted the time needed for cells labeled in late S-phase to traverse into mitosis. Pattern 1 was observed more often at 4 h after EdU incorporation, indicating that such labeling was typical for late S-phase. (PDF 147 kb)

Additional file 3: Figure S3. Distribution of EdU labeled regions in *N. damascena* metaphase chromosomes (pattern 1). (PDF 378 kb)

Additional file 4: Figure S4. Estimation of chromosome and chromatin fiber widths (longitudinal section of telophase chromatid). The typical cross-sections of telophase chromatid and/or chromatin fibrils are indicated with colored lines (black – chromatid, blue – ‘300 nm fiber’, green - chromonema). Scale bar: 2 μm. (PDF 2057 kb)

## Abbreviations

DAPI: 4′,6-diamidino-2-phenylindole
EdU: 5-ethynyl-2′-deoxyuridine
*N. damascena*: *Nigella damascena* L.
PALM: Photoactivated localization microscopy
