# Supplementary material for: Visualization of chromosome condensation in plants with large chromosomes
Source: BMC Plant Biol. 2017 Sep 12;17:153. doi: 10.1186/s12870-017-1102-7 (PMC5596468; doi:10.1186/s12870-017-1102-7)
Supplement: Supplementary file 1 — Chromosome organization in plants with different genome sizes. a Cucurbita pepo; b Brassica rapa; c Lupinus polyphyllus; d Lycopersicon esculentum; e Vicia sativa; f Hordeum vulgare; g Rudbeckia hirta; h Lathyrus odoratus, i Lathyrus latifolius; j Allium porrum. Axial chromatin-free cavities are indicated by arrows. For each plant species, several anaphase and telophase cells were analyzed, and the image of the most representative cell is shown. Scale bars: 0.5 μm. (PDF 3315 kb) [file 12870_2017_1102_MOESM1_ESM.pdf]

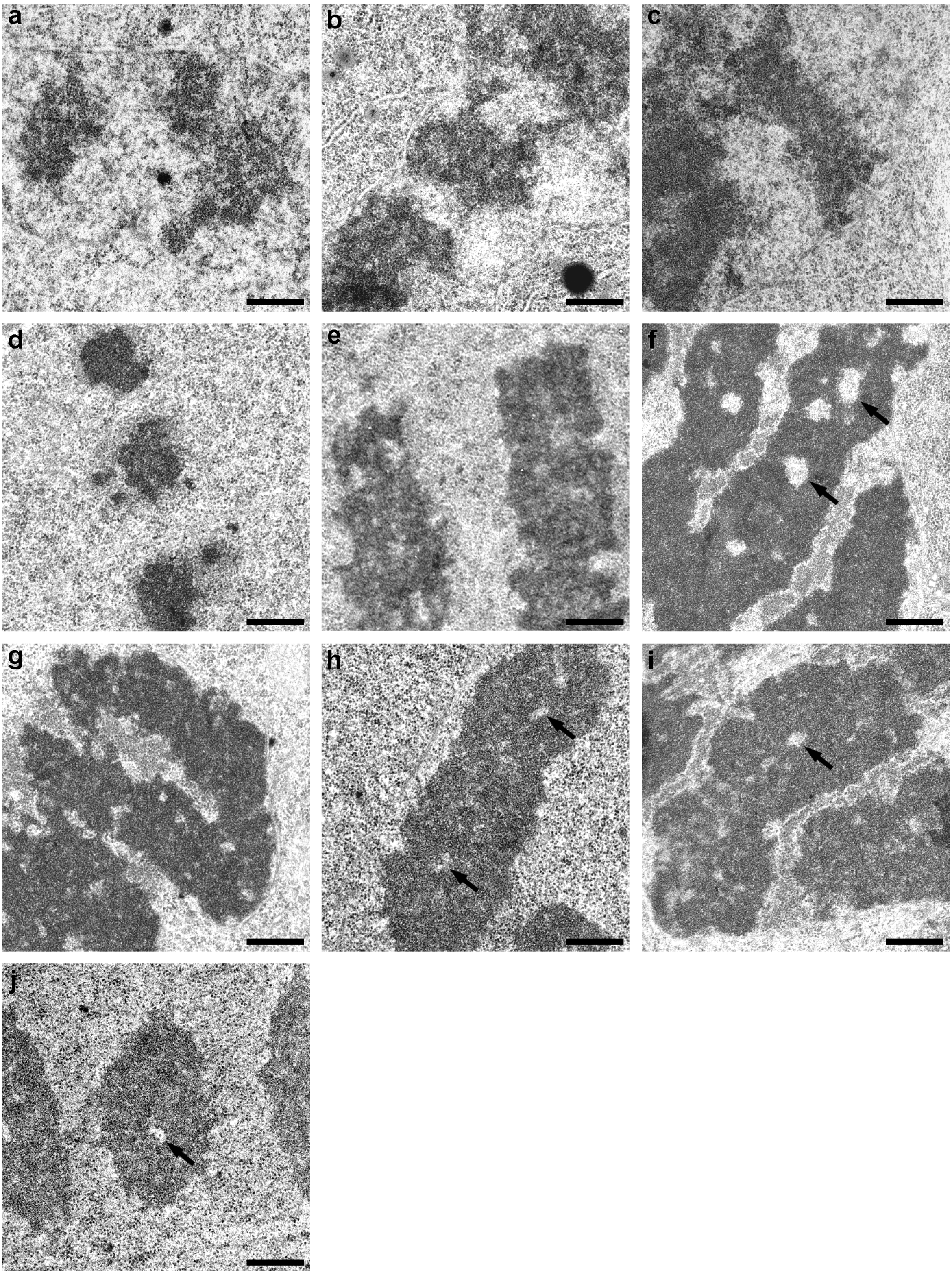

Figure S1 Chromosome organization in plants with different genome sizes. **a** *Cucurbita pepo*; **b** *Brassica rapa*; **c** *Lupinus polyphyllus*; **d** *Lycopersicon esculentum*; **e** *Vicia sativa*; **f** *Hordeum vulgare*; **g** *Rudbeckia hirta*; **h** *Lathyrus odoratus*, **i** *Lathyrus latifolius*; **j** *Allium porrum*. Axial chromatin-free cavities are indicated by arrows. For each plant species, several anaphase and telophase cells were analyzed, and the image of the most representative cell is shown. Scale bars: 0.5 μm.
