# Supplementary material for: Visualization of chromosome condensation in plants with large chromosomes
Source: BMC Plant Biol. 2017 Sep 12;17:153. doi: 10.1186/s12870-017-1102-7 (PMC5596468; doi:10.1186/s12870-017-1102-7)
Supplement: Supplementary file 2 — Asynchronously growing cells of N. damascena roots were pulse labeled with EdU for 30 min, incubated in 200 μM thymidine for 30 min, and then the incubation was continued in the distilled water (i.e., in the absence of EdU). The brief pulse labeled all S-phase cells, and the initial appearance of EdU-labeled mitotic figures thus denoted the time needed for cells labeled in late S-phase to traverse into mitosis. Pattern 1 was observed more often at 4 h after EdU incorporation, indicating that such labeling was typical for late S-phase. (PDF 147 kb) [file 12870_2017_1102_MOESM2_ESM.pdf]

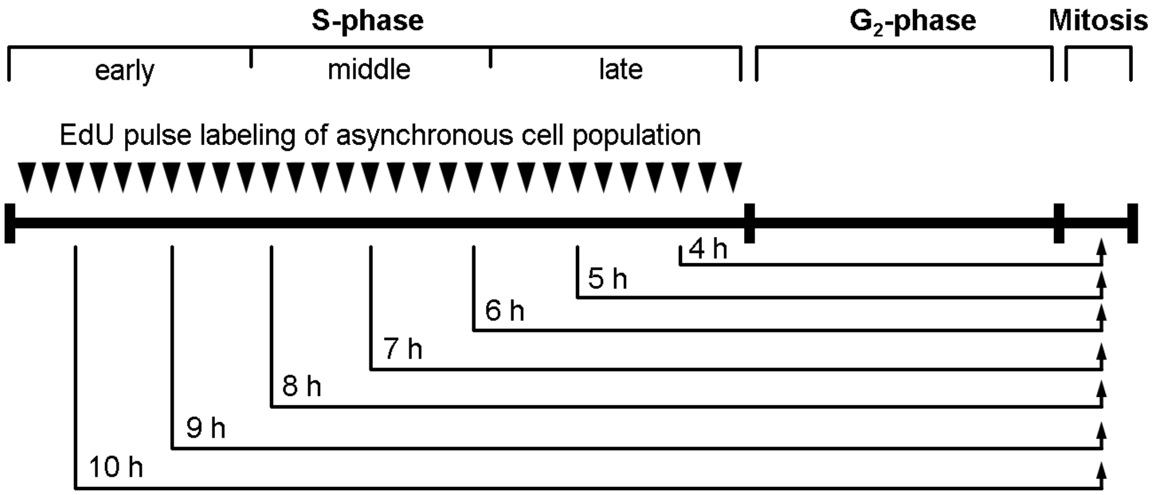

Figure S2 Asynchronously growing cells of *N. damascena* roots were pulse labeled with EdU for 30 min, incubated in 200 µM thymidine for 30 minutes, and then the incubation was continued in the distilled water (i.e., in the absence of EdU). The brief pulse labeled all S-phase cells, and the initial appearance of EdU-labeled mitotic figures thus denoted the time needed for cells labeled in late S-phase to traverse into mitosis. Pattern 1 was observed more often at 4 h after EdU incorporation, indicating that such labeling was typical for late S-phase.
