# Supplementary figures and images for: Visualization of chromosome condensation in plants with large chromosomes
Source: BMC Plant Biol. 2017 Sep 12;17:153. doi: 10.1186/s12870-017-1102-7 (PMC5596468; doi:10.1186/s12870-017-1102-7)

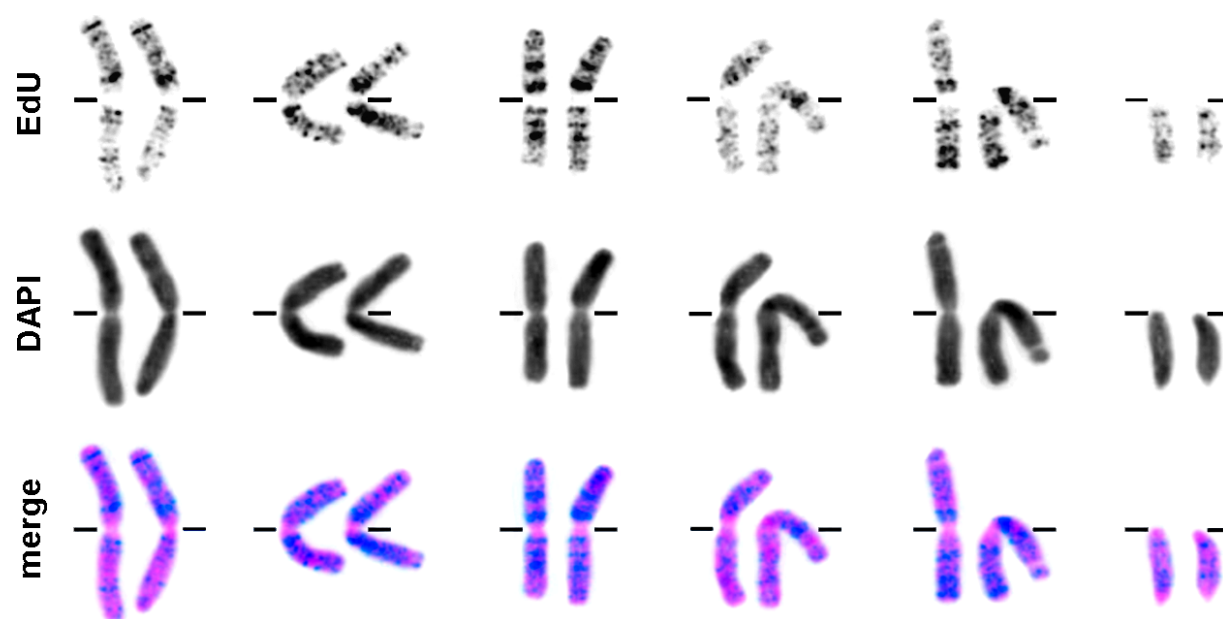

**Figure S3** Distribution of EdU labeled regions in *N. damascena* metaphase chromosomes (pattern 1).

Supplement: Supplementary file 3 — Distribution of EdU labeled regions in N. damascena metaphase chromosomes (pattern 1). (PDF 378 kb) [file 12870_2017_1102_MOESM3_ESM.pdf]
