# Supplementary material for: Visualization of chromosome condensation in plants with large chromosomes
Source: BMC Plant Biol. 2017 Sep 12;17:153. doi: 10.1186/s12870-017-1102-7 (PMC5596468; doi:10.1186/s12870-017-1102-7)
Supplement: Supplementary file 4 — Estimation of chromosome and chromatin fiber widths (longitudinal section of telophase chromatid). The typical cross-sections of telophase chromatid and/or chromatin fibrils are indicated with colored lines (black – chromatid, blue – ‘300 nm fiber’, green - chromonema). Scale bar: 2 μm. (PDF 2057 kb) [file 12870_2017_1102_MOESM4_ESM.pdf]

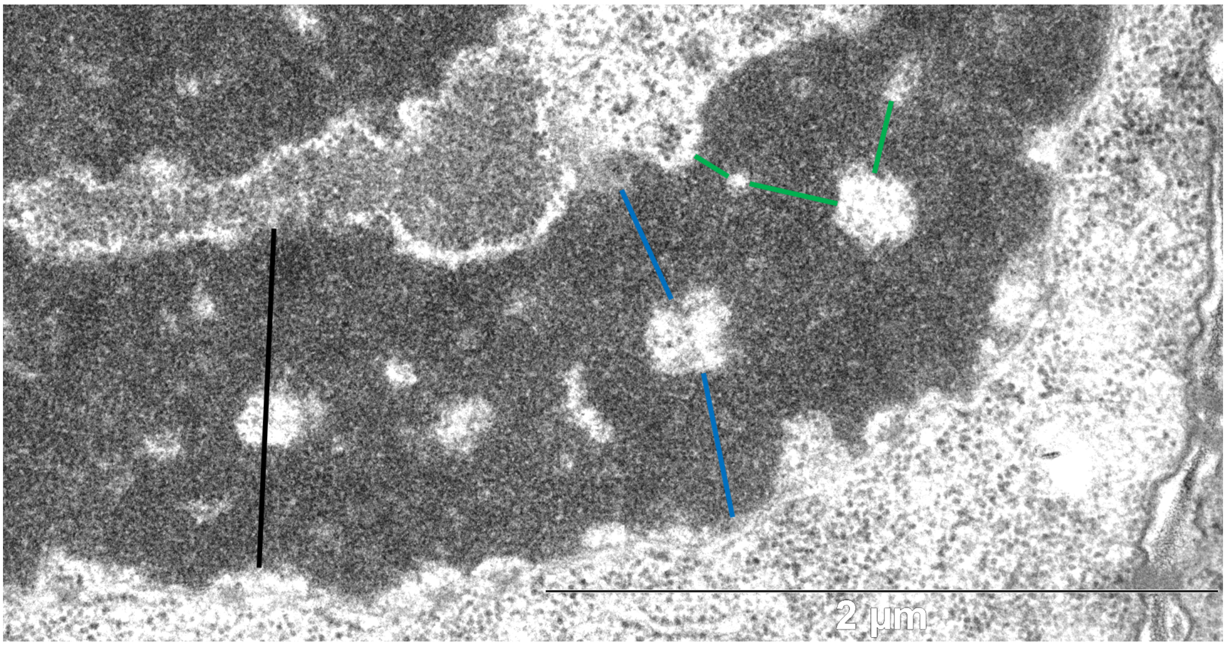

Figure S4 Estimation of chromosome and chromatin fiber widths (longitudinal section of telophase chromatid). The typical cross-sections of telophase chromatid and/or chromatin fibrils are indicated with colored lines (black – chromatid, blue – '300 nm fiber', green - chromonema). Scale bar: 2 µm.
